# Veterinarian burnout demographics and organizational impacts: a narrative review

**DOI:** 10.3389/fvets.2023.1184526

**Published:** 2023-07-04

**Authors:** Michele A. Steffey, Dominique J. Griffon, Marije Risselada, Valery F. Scharf, Nicole J. Buote, Helia Zamprogno, Alexandra L. Winter

**Affiliations:** ^1^Department of Surgical and Radiological Sciences, School of Veterinary Medicine, University of California, Davis, Davis, CA, United States; ^2^Western University of Health Sciences, College of Veterinary Medicine, Pomona, CA, United States; ^3^Department of Veterinary Clinical Sciences, College of Veterinary Medicine, Purdue University, West-Lafayette, IN, United States; ^4^Department of Clinical Sciences, North Carolina State University College of Veterinary Medicine, Raleigh, NC, United States; ^5^Department of Clinical Sciences, Cornell University College of Veterinary Medicine, Ithaca, NY, United States; ^6^Evidensia Oslo Dyresykehus, Oslo, Norway; ^7^Merck & Co., Inc., Rahway, NJ, United States

**Keywords:** veterinary, burnout, occupational stress, wellbeing, practice management

## Abstract

Burnout is a work-related syndrome of physical and emotional exhaustion secondary to prolonged, unresolvable occupational stress. Individuals of different demographic cohorts may have disparate experiences of workplace stressors and burnout impacts. Healthcare organizations are adversely affected by burnt out workers through decreased productivity, low morale, suboptimal teamwork, and potential impacts on the quality of patient care. In this second of two companion reviews, the demographics of veterinary burnout and the impacts of burnout on affected individuals and work environments are summarized, before discussing mitigation concepts and their extrapolation for targeted strategies within the veterinary workplace and profession.

## 1. Introduction

The pathogenesis of burnout was explored in the first part of these two companion reviews (1), placing an emphasis on the pathophysiology of the syndrome and resultant impacts to individual veterinarians and other health care providers. This second part of the topic review focuses on the epidemiology of burnout and its consequences within the workplace, including effects on patient care, client satisfaction, teamwork, staff turnover, and other practice logistics (2–4). Human resources are the most important assets of any organization, and conditions that lead to clinician burnout will ultimately impact the functionality of the affected practice or team. Burnout is a deep-rooted, self-perpetuating occupational problem whose resolution will require changes in professional culture, workplace climate, and in some instances, organizational structure, and function.

## 2. Burnout demographics

### 2.1. Students

At the beginning of their medical training, student physicians report significantly less depression and burnout and a better quality of life than other college-educated peers, but notably their wellbeing decreases during their 4 years of medical education (5). Approximately 44% of medical students experience burnout (6). Up to 28% of medical students experience depression, compared to 8% of the general population (7). In medical students, factors within the learning and work environment, rather than individual attributes, are the major drivers of burnout (8). Learning environment factors that lead to burnout include disorganized rotations and inadequate supervision, high workload, the stress of tests, and chronic, maladaptive, short-term goal-driven studying and living patterns (8).

Similarly, 32% of 1st year veterinary students experience clinical levels of depressive symptoms and report higher anxiety levels than medical students and the U.S. general population (9, 10). First and 2nd year veterinary students experience moderate feelings of burnout and the greatest levels of emotional exhaustion during the spring semesters (11). Over the course of the 3 pre-clinical years, veterinary student empathy declines, and personal distress rises (12). In general, veterinary students report high levels of burnout, poor mental health, and good physical health (13). Risk factors for poor mental health in veterinary students include perceived poor physical health, unclear expectations in the curriculum, difficulty fitting in with peers, excessive academic workload, and homesickness (11, 13). Additional reported stressors include unsatisfactory family and personal relationships, debt and financial self-insufficiency, lack of time for social and recreational activities, chronic sleep deprivation, time demands, the experience of constant academic evaluation, and academic concerns (11, 13).

In spite of these documented stressors and effects on mental health, a large proportion of affected students do not seek help, citing barriers such as fear of disclosure, documentation, or unwanted intervention, and lack of time (14). Stigma and self-stigma to burnout and a range of mental health issues including stress, depression and suicidal ideation have been reported among medical and veterinary students (15–20). Recent research revealed that a large proportion of medical students that identify a need for mental health support do not seek help or use available services (21, 22). Approximately 50% of medical students perceived that residency program directors, supervisors, peers, and patients held negative attitudes about mental illness and its treatment (22).

Personal distress can secondarily lead to unprofessional behaviors and attitudes, even when students feel guilty about engaging in such behaviors, and medical professionalism attributes deteriorate as mental wellbeing issues grow (23–25). Burnout in medical students was associated with self-reporting ≥1 unprofessional behaviors (OR = 1.76, 95% CI = 1.45–2.13) or holding less altruistic views regarding physicians' responsibility to society (OR = 1.65, 95% CI = 1.35–2.01) (26). Healthcare students acquire professional values and behaviors from informal observation of mentors and role models, a concept termed the “hidden curriculum” (27). Expectations of medical culture are generally that “good doctors” do not complain, shirk work, or exhibit pain, distress, or symptoms of mental illness (28). The formal educational culture may advocate teamwork and professionalism, but the hidden curriculum tends to incentivize performance and competitiveness over collaboration, which may lead to cynicism (29, 30). Conflicts between the hidden curriculum and an outwardly proclaimed prioritization of wellbeing manifest and create dilemmas for trainees when the behaviors that they observe from mentors are at odds with their understanding of best practices, and the hidden curriculum can cause an increase in acceptance of unethical behaviors (31, 32). Poor self-treatment by teachers and mentors sends powerful messages to students, who internalize maladaptive concepts of professionalism, adopt similar behavioral patterns, and then perpetuate this messaging themselves (29). Veterinary and medical school set the stage for later professional burnout, which is viewed by some as an inevitable consequence of the way healthcare education is structured (29).

### 2.2. House officers (interns/residents/clinical fellows)

Veterinary house officers experience high levels of burnout, characterized by high emotional exhaustion and low personal accomplishment (33). The mental component of their quality-of-life scores scored consequently lower than the general US population (33). Similarly physician house officers are at increased risk for burnout and psychological morbidity, and report a poorer quality of life than their attendings (34). In a study of 91 emergency physicians at 2 teaching hospitals, at least half suffered from burnout as early as their 2nd year of residency training, with residents more likely to report higher scores on the depersonalization subscale (74% residents vs. 39% attendings, *p* < 0.011), to screen positive for depression (48 vs. 19%, *p* < 0.012) and to report lower quality of life scores (6.7 vs. 7.4 on a scale of 10, *p* < 0.036) than attendings (35). In a study of 247 physician interns, telomeres (cellular indicators of aging) were found to shorten five times as much during an internship as during a typical year of life (*p* = 0.008) (36). In the same study, the magnitude of attrition correlated with workload (*p* = 0.002) (36). These results provide measurable and alarming indicators of the physiologic toll that current methods of training impose on clinicians. In a study of 582 physician surgical residents, 22% screened positive for workplace PTSD, and an additional 35% were at risk (37). For another cohort of physicians, working an average of >69 h/week (*p* < 0.001), screening as high risk for burnout (*p* < 0.001), and feeling unhealthy (*p* < 0.001) were all risk factors associated with a diagnosis of PTSD (37). Burnout and low quality of life in healthcare not only affects the individual, but also their loved ones. Spouses of resident surgeons notably exhibited similar rates of psychological distress and burnout as their partners (38).

Sources of resident stress include work overload, financial instability, insufficient collegiality, inadequate mentoring from supervising faculty, employment opportunity concerns, difficult client interactions, poor mental health, high academic demands, student teaching expectations, staff expectations, time constraints for specialty examination preparation, the process of learning advanced clinical skills, constant performance evaluation, and expectations to design and perform clinical research under programmatic time constraints (33, 39). Veterinary house officers commonly describe an inability to balance training demands with maintenance of at least one aspect of personal health such as exercise, social engagement, diet, and economic satisfaction, and more than half of house officers report their work-life balance as unsustainable (40). Almost 1/3 of participating veterinary house officers evaluate their current eating habits as poor, with >90% attributing this at least partly to programmatic demands (40). Veterinary residents in academia are particularly affected as they receive 2–3 fewer days off per month and obtain 3–4 h less sleep per 48 h than those in private and corporate practice (41). Inpatient volume is a major component of resident workload (42). Similar to trends felt by veterinarians, physicians also describe that the pace, complexity, and intensity of inpatient care has increased with time, which challenges the ability to emphasize education for both house officers and students in teaching hospitals (43–45).

Resident physicians that meet the criteria for burnout report inadequate sleep and frequent extended shifts as major stressors (46). Irrespective of their year of training, all resident physicians reported poor sleep quality and highly variable duration of sleep (by +/– a mean of 2.9 h, SD = 1.4) even if their duration of daily sleep over a week averaged 7 h (SD = 1.8) (47). Residents covering more than six 24-h on-call shifts/month were 10-times more likely to have poor sleep quality (OR = 10.4; 95% CI = 2.2–48.7, *p* < 0.003) (48). After working 30-h shifts, resident physicians exhibited elevated serum levels of cytokines and inflammatory markers at levels that may lead to vascular injury and atherosclerosis with repeated sleep deprivation (49). Sleep quality and fatigue do not rebound to more acceptable levels as residency progresses (47). Veterinary residents reported a weeknight mean of 6.0 h/night (range, 5.5–7.9, varied by specialty) and weekend mean of 6.6 h/night (range, 5.8–7.5) of sleep; similar to findings in resident physicians, the number hours slept was not associated with year in training (50). Veterinary residents who reported sleeping an average of 4–5 h/night when on clinics were more likely to describe their caseload as too high (OR = 4.84; 95% CI = 2.74–8.56; *p* < 0.001) (50). High resident workload is associated with decreased participation in educational activities and increased fatigue-related medical errors (34, 44). Self-perceived medical errors were associated with a subsequent decrease in quality of life (*p* < 0.02) and worsened measures in all three burnout domains (p < .002 for each) by resident physicians (51). Forty percent of veterinary residents in a recent survey expressed concerns over making major medical errors (41). Given that a recent study of more than 300 veterinary house officers reported that the majority (58%) spend 11–13 h in the clinic on an average weekday, and that they reported to the clinic 5 days (33%), 6 days (34%), or 7 days (32%) per week (implying average workweeks of 55–91 h/week), it is unsurprising that the profession is seeing manifestations of severe stress in veterinary trainees (50). An argument can be made for high caseloads to improve training by broadening exposure and allowing repetitive practice. However, these advantages must be balanced against the ethics of patient safety, the negative impacts of fatigue on learning and individual health, and the reduced opportunities for didactic study that may counteract the benefits of increased case exposure (52–54). In graduate students, improved sleep duration and sleep quality moderates the relationship between stress and the exhaustion dimensions of burnout (55).

Most veterinary house officers consider their current economic situation to be fair or poor, and a striking 95% report feelings of anxiety related to finances (40). This finding likely reflects that veterinary resident and intern annual salaries do not meet the minimum income standard of a living wage (56). Salary gaps between residency programs also exist, with reported salaries of $40,000 ± $10,000 per year in academic programs, and about $10,000/year more in private practice residencies (56). By comparison, new graduates hired as full-time associate veterinarians reported a mean private practice starting salary of $111,242 in 2022 (57). On top of these pay disparities, 87% of veterinary graduates leave school with veterinary degree student loan debt (58). Inadequate financial reward for work and financial stresses are consistently demonstrated to be important contributors to burnout among veterinary and physician house officers (40, 59, 60).

### 2.3. Personal demographics

The primary risk factors associated with burnout are generally similar among all healthcare professionals, but differences between subgroups justify discussion. While burnout is an important problem for all physicians, a recent meta-analysis found that the prevalence of burnout was higher in women than in men in almost 90% of studies comparing physician burnout between genders (61). The relative risk of any burnout in women physicians is approximately double that of men physicians (OR = 1.97; 95% CI = 1.2–3.4) (62). Women physicians are more likely to suffer from emotional exhaustion whereas men are more likely to describe depersonalization as manifestation of burnout (63). These findings logically lead to questioning whether burnout is more common among women healthcare professionals, or if gender-based differences in the experience and expression of burnout make its occurrence easier to identify in women (34). Evidence suggests that women physicians may have different occupational experiences than men in the same positions, driven by unequal patient expectations, different experiences within the workplace, and role expectations outside of work (61, 64–67).

Differences in the experience of the workplace and in the importance of various job demands, societal experiences, and personal and professional resources for men and women result in differential stressors and effects of the workplace (68). Employed women with working partners still perform an additional 2 h of domestic work per day on average, an amount three times higher than that reported by men with working partners (69–71). Compared to men in the same profession, women physician surgeons were more likely to believe that child-rearing had slowed their career advancement (57 vs. 20%, *p* < 0.001), to have experienced a conflict with their partner's career (53 vs. 41%, *p* < 0.001), or to have experienced a recent work-home conflict (62 vs. 49%, *p* < 0.001) (72). Women healthcare professionals are more likely to face both conscious and unconscious gender biases and discrimination, a lack of parity in salaries, and greater expectations for deferred personal life decisions, as well as experience disproportionate impacts of childbearing and childrearing, greater challenges associated with dual-career couple status, and face additional barriers to professional advancement (including reduced opportunities for mentorship and networking) (61, 65, 73).

Despite similar work and levels of productivity among academic physicians, women are less likely to describe supportive environments or manageable work conditions, to feel a sense of common purpose and belonging within the organization, to report fair access to opportunity and rewards, and are less likely to be promoted than men (74–78). In a study of 3,648 evaluations of 1,066 physician faculty, women scored lower than men independent of performance (*p* < 0.001) (79). In a study of 2,013 entries submitted to a single hospital management portal, women were more frequently criticized for lack of communication (*p* < 0.002) whereas complaints about men were more often related to perceived medical errors (*p* < 0.02) (80). In these complaints, women were more likely than men to be criticized for violating their perceived station in the social hierarchy with adjectives including “insensitive,” “arrogant,” “demeaning,” and “condescending” (80). Women physicians who deviate from gender stereotypical behaviors are often considered unprofessional (80), and it is theorized that this phenomenon is especially pronounced in fields that are historically male-dominated (79–81). Regarding women as unprofessional on the basis of non-adherence to gender stereotypes is likely to contribute to burnout in female physicians (82). Distorted gender-specific perception of clinicians may underwrite appraisals of clinical competence or lack thereof, salary discrepancies, and inequitable promotion criteria. Women academic physicians overall experience more gender bias in their careers (66%, 95% CI = 62–70%) compared to men (10%, 95% CI = 8–13%) and were more likely to report having personally experienced sexual harassment (30%, 95% CI = 26–35% vs. 4%, 95% CI = 3–6%) (83). Similarly, work-driven PTSD tends to be more common in women physicians and women serving in the military compared to male counterparts (37). These studies all describe high levels of chronic occupational stress that contributes to burnout.

Approximately 50% or more of veterinarians report moderate to high levels of burnout (84, 85) and in general, veterinarians exhibit similar overall demographics as physicians: women veterinarians exhibit higher burnout risks and scores compared to men (4, 85, 86), younger/less experienced veterinarians exhibit higher burnout rates compared to those with more experience (85) and greater burnout risk is seen among those carrying higher educational debt loads (85). While historically veterinary medicine has been a male-dominated profession, women currently account for ~80% of graduating veterinarians in the U.S. each year and represent an increasing proportion of practicing veterinarians at 64% of the U.S. veterinary workforce in 2020 (87, 88). Despite the rising proportion of women in veterinary medicine over the past decades, similar gender disparities to those described for physicians still exist. Women veterinarians are less likely to be paid equivalently, to be practice owners, to exhibit the same progression through academic ranks as men, or to be in professional leadership positions (89–94). Among veterinary surgeons, the personal income of men was a mean of 18% greater (95% CI = 12–25; *p* < 0.001) than that of women (89), and in academia, male veterinary surgeons were more likely than women to be associate or full professors (OR = 2.52, 95% CI = 1.03–6.14, *p* < 0.042) (90). Women veterinarians remain underrepresented on editorial boards including managing editors (32% women vs. 67% men) and editors (35% women vs. 65% men) (95). Working veterinarian mothers reported high rates of perceived maternal discrimination (73%) or workplace inequity (58%) (96). Perceptions of work relationships and support impact women veterinarians' job satisfaction more than that of men's (97). Women veterinarians continue to face demonstrable discrimination in the workplace, which may help explain why satisfaction with workplace support is more strongly linked positively with resilience and negatively with burnout in women veterinarians (4, 98, 99).

While evidence about veterinary burnout is growing, its impact on many demographic subgroups in the profession remains poorly documented. However, a lack of diversity, equity, and inclusiveness in the veterinary profession has been reported as a workplace stressor (100). A recent meta-analysis in physicians only identified nuances and trends between underrepresented minorities and non-underrepresented minority physicians, recommending further studies (101). Medical students in sexual minority groups experience increased symptoms of burnout compared to heterosexual students with evidence of differences in both disengagement and exhaustion; similar impacts have been seen in practicing physicians (102). LGBTQIA+ individuals experience more psychological distress, suicidal ideation and suicide attempts in school and as veterinary and medical professionals (99, 103, 104). Discrimination and harassment in the workplace of any kind can lead to feelings of isolation, an important risk factor for burnout (105).

Age has been associated with burnout as an independent variable, with younger physicians and veterinarians being at greater overall risk than their older counterparts (34, 106–108). This finding likely reflects greater professional uncertainties and less professional confidence in junior healthcare providers, combined with student debt and financial uncertainties (107, 109). However, interpretation of these statistics should also consider survival bias, as burnt-out clinicians are more likely to change careers and be excluded from the sampled population (34). Conversely, age influences resilience to fatigue, a large risk factor for burnout; objective performance measures decline under conditions of fatigue more steeply from the age of 35 upwards (110). Older physicians seem to be especially vulnerable to stress factors impacting unhappiness with on-call work, whereas younger physicians and specialist trainees seem vulnerable to dissatisfaction with on-call work relative to job resources (111).

Without minimizing the impacts of burnout to any individual veterinary professional and while emphasizing best practices for all, it is also important for the profession to be aware of differential work experiences, as well as differences in perception of burnout, identification of burnout, and in the factors contributing to the development of burnout for individuals of different backgrounds (112). The risk factors, prevention strategies, intervention initiatives, and outcomes of professional burnout may differ for women, underrepresented minority and LGBTQIA+ professionals compared to the historical majority population of veterinary clinicians. Recommended interventions for burnout risks may be prioritized differently by underrepresented professionals, and in general, organizations must continue to address the pervasiveness of unconscious and explicit biases that create disparities of experience at work that contribute to burnout (65, 112). Optimal interventions may also be different for professionals at different life stages or in different professional positions (e.g., residents vs. attendings, practice owners vs. associates, large animal vs. small animal focus, general vs. specialty practice). Awareness of these differences is required in order to better understand and address the full scope of professional burnout.

## 3. Effects of practice type

### 3.1. Clinical practice

Forty-four percent of private veterinary practitioners report considering leaving the profession, including >40% of those who graduated within the last 10 years (113). Self-employed veterinarians express stress more frequently but also a better morale than associate veterinarians (107). Associate veterinarians are twice as likely to develop feelings of reduced job satisfaction and are also more likely to experience burnout relative to practice owners (91, 114). While both experience stress, veterinary practice owners exhibit far less burnout than non-owner associates (115). These findings are concerning as the rate of veterinary practice ownership has declined from 45% in 2013 to 36% in 2020 (114). Increasing numbers of veterinary practices have been purchased by consolidating corporations where clinicians have no ownership stake and reduced decision-making input, an organizational structure that may promote work stress and burnout (1, 116–119). Amongst physicians, burnout was greatest and job satisfaction was poorest in hospital settings (OR = 1.88, 95% CI = 0.91–3.86), those aged 31–50 years (OR = 2.41, 95% CI = 1.02–5.64), and among those working in emergency medicine and intensive care (OR = 2.16, 95% CI = 0.98–4.76) while burnout was lowest among general practitioners (OR = 0.16, 95% CI = 0.03–0.88) (120). Still, general practice generates sufficient stress levels to prompt increasing numbers of physicians to leave traditional practices for concierge medicine (121). Anecdotally, similar shifts away from traditional practice models are beginning to be seen in veterinary medicine, with increasing numbers and types of non-traditional types of clinical veterinary practice including shelter medicine, house call general practice, high volume spay/neuter practice, locum service-based practice, mobile imaging or surgery specialists, and remote specialty consultation-based practice (including diagnostic imaging, internal medicine, pathology, and clinical pathology specialists). Although data on the impact of specialization on burnout is limited in veterinary medicine, veterinarians primarily engaged in companion animal practice exhibit higher burnout scores than veterinarians in other types of clinical practice (85, 122–124). Certain subsets of veterinary practice (such as shelter medicine) are also more likely than others to experience particular forms of work stress related to empathic distress, secondary trauma and moral injury that can also subsequently contribute to burnout (125–130).

### 3.2. Academia and other professional sectors

While data are limited, in a study of occupational health across veterinarians with a wide range of professional focus in addition to clinical practice, those in education and research reported the highest levels of stress, and in another study, those in research, teaching, industry and government positions experienced the highest levels of depression (124, 131). Seventy percent of academic veterinarians reported depressive symptoms in the 2 weeks prior to a 2021 survey (41). A recent survey found that 62% of the veterinary faculty studied met the criteria for burnout, with overall burnout scores higher than a reference group of academic physicians (*p* = 0.027) (132). Veterinary academicians report working substantially more hours and receiving fewer days off per month than private practitioners (41). Private practice small animal surgeons reported working 40–49 h/week compared with 50–59 h/week by academic surgeons (*p* < 0.001) (89). Burnout syndrome in academic clinicians is associated with psychological implications, disengagement, and reduced confidence (133).

Serious time constraints and competing priorities are compounded by task complexity and time-consuming logistics associated with wide-ranging academic duties. The duties of academic clinicians most commonly include patient care and clinical instruction in teaching hospitals. Veterinary teaching hospitals increasingly manage a tertiary referral caseload, with high case complexity, urgency, and risks of complications, higher communication load, and more complex practical logistics. The inherent high complexity and low job predictability increases cognitive load, which can in turn affect clinical performance, patient safety and lead to burnout (134). Additionally, the incentives in modern veterinary academic practice are consistently misaligned with stated goals. Complex practices such as teaching hospitals require teamwork; however, prestige, funding, time and resource pressures, and a winner-take-all mentality incentivize individualistic behaviors over team-promoting ones.

In addition to clinical stressors, academic veterinarians face academia-specific workplace stressors, including the pressures of scholarly expectations, administrative contributions, didactic teaching loads, responsibility for mentorship and supervision of clinical trainees, insufficient reward, diminishing institutional resources, and academic bullying (135). In a literature review of faculty experiences with bullying in higher education, the prevalence of bullying ranged between 18 and 32% (136). Professional autonomy coupled with expectations for high performance assessed with subjective and peer evaluations can all trigger bullying behavior among faculty (137). However, ambiguous identification of bullying behaviors, low-to-absent reporting, and non-standardized repercussions creates a culture and environment in which bullying is tolerated and perpetuated (138).

Increased research productivity and teaching load expectations have resulted in unrealistic workloads and deterioration in the quality of workplace relations (137). The need to expand sources of revenues in a competitive environment without compromising complex accreditation standards has increased the levels of stress placed on students and faculty (76, 137). Emotional exhaustion and burnout may influence not only the delivery of quality clinical care, but also the quality of training provided to students and house officers (133, 139). The diversity of expectations and responsibilities placed on clinicians in academia frequently leads to role conflict or ambiguity, both sources of work-related stress, reduced productivity, and impaired organizational efficiency, thereby correlating with burnout (140). Insufficient focus or flexibility in assignment of effort within academic positions to account for personal strengths, passions, knowledge, and skills exacerbates existing issues of work stress. From this standpoint, “*to have fixed expectations of all faculty, without accounting for individual needs, is a recipe for failure. There are preferences which should, ideally, be met in order to best allow for a fulfilling career and a highly functioning and sustainable group. If we force our own definition of career onto others, this might contribute to depression and burnout*” (141). But accommodating faculty's personal interests and needs creates challenges for veterinary colleges, as they balance education, research, and service missions while facing dwindling state support and competition with the private sector for the caseloads needed for training purposes. In general, veterinary faculty value their work and their patients, but face excessive workloads and lack the autonomy to make changes (132).

## 4. Impacts of professional burnout on the organization and management considerations

### 4.1. Teamwork and climate

**Figure 1:**
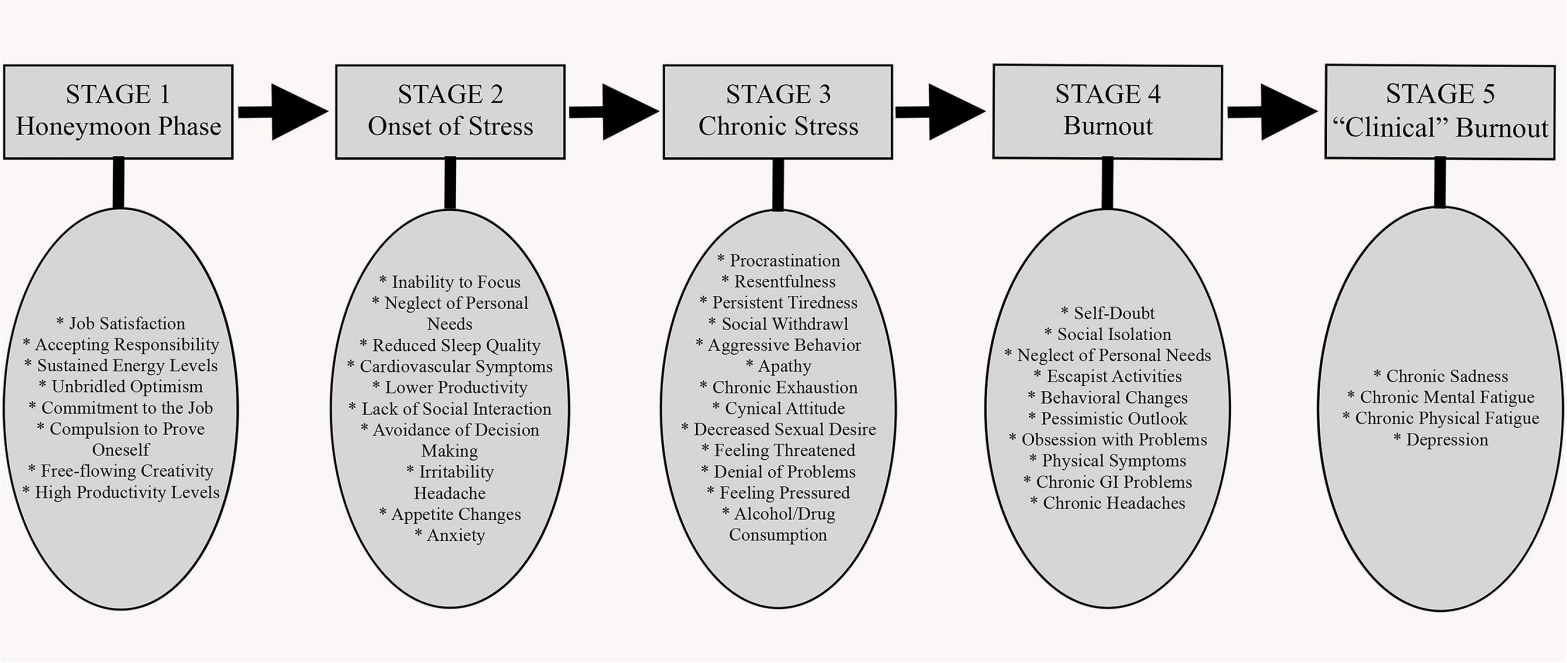
The progression of burnout in an individual employee; individual and organizational impacts. Adapted from information presented by de Hert (142).

Occupational stress is perceived when occupational stressors tax or exceed an individual's ability to cope, resulting in consistent, predictable physiological and behavioral outcomes that ultimately impact the workplace (Figure 1) (117, 142). Affected clinicians who are not able to either address the underlying stressors or to physically withdraw from practice may eventually be unable to modulate their wellbeing, affect, and energy in both verbal and non-verbal behaviors, and commonly will psychologically withdraw (118). Physically present but psychologically withdrawn, a burnt-out clinical workforce will have significant adverse effects on the veterinary business itself, with direct and indirect fiscal impacts, morale erosion, toxic work environments and disrupted teams, impacts to practice reputation, low productivity, high rates of absenteeism and staff turnover, increased medical error rates, and reduced client satisfaction (65, 118). Challenges in balancing personal life with work are clearly associated with burnout (143, 144). Work-life integration is typically considered an individual variable, but notably this factor actually operates at the workplace climate level and a positive work-life climate is consistently associated with better teamwork, increased safety and lower burnout (*p* < 0.001) (145). Importantly though, it should be noted that healthcare workers only report comfort in tending to their non-work needs when institutional cultural norms, supervisors, and coworkers also genuinely demonstrate a commitment to work-life integration themselves (146).

### 4.2. Patient care

Evidence suggests that clinician burnout impacts patient care independent of clinical experience (35). Burnout-affected physicians consistently exhibit twice the risk of having patient safety incidents as those unaffected: (OR = 2.04, 95% CI = 1.69–2.45) (120) and (effect size = 2.67, 95% CI = 2.3–3.0) (147). In a study of nearly 8,000 physician surgeons, burnout (OR = 2.02, *p* < 0.0001) or depression (OR = 2.22, *p* < 0.0001) independently predicted self-reporting a recent major medical error (148). In another, burnout-affected physicians were more likely to self-report one or more suboptimal patient care practices each month (53 vs. 21%; OR = 8.3, 95% CI = 2.6–26.5, *p* = 0.004) (149). Each one-point increase in depersonalization (scale range, 0–33) was associated with an 11% increase in the likelihood of reporting an error, while each one-point increase in emotional exhaustion (scale range, 0–54) was associated with a 5% increase (148). Surgeons reporting errors worked an average of 4.6 more hours/week (63.5 vs. 58.9 h; *p* < 0.0001) and spent an additional hour/week in the operating room (18.2 vs. 17.1 h; *p* < 0.01) than those not reporting errors (148). Medical errors in a group of 6,586 physicians were more likely to be reported by physicians affected with burnout (OR = 2.22, 95% CI = 1.79–2.76) or fatigue (OR = 1.38, 95% CI = 1.15–1.65) (2). Conversely, reporting a medical error is associated with measurable effects on burnout and mental health in medical professionals, including a >1 standard deviation decline in mental quality of life score (a difference reported to be clinically significant), measurable increases in all 3 domains of burnout, and roughly a doubling in the risk of screening positive for depression (54.9 vs. 27.5%; *p* < 0.0001) (148). Stress, illness, and fatigue have also been identified as causes of error in veterinary practice (150). A reciprocal cycle is created in which burnout-affected individuals are more likely to report having caused a medical error, and having contributed to a medical error is a stressor associated with the development or worsening of burnout (51). An individual experiencing burnout may not only be unable to maintain appropriate patient and workplace safety, but may continue to further deplete their personal mental, physical, and emotional reserves in attempting to do so (134).

### 4.3. Fiscal impacts of burnout

Clinician burnout generates organizational costs through absenteeism, clinician turnover, signing bonuses, and ramp-up costs for new hires. Reduced organizational effectiveness compounds this effect through lost patient capacity and billings as a result of reduced working hours, lost tacit knowledge, mentorship, work routines, internal, and external relationships (123). Employers can benefit from optimizing work-life integration through cost savings, improved recruitment and retention, job satisfaction and commitment, improved quality of the workforce, and more positive employee attitudes (145). Client satisfaction is another factor that may contribute to fiscal consequences of burnout for a practice. Similar associations have been reported between physician burnout score and patient satisfaction and between veterinarian mental health measures and client satisfaction scores (151).

At best, individual recovery from severe burnout may be prolonged, requiring months away from work (152). Whereas, 80% of employees with serious but short-term stress recover fully within weeks, the recovery from severe burnout usually requires a work hiatus that may take more than a year, and even 2–4 years later 25–50% of such impacted individuals are not fully recovered (153). This is profoundly concerning at both individual and organizational levels when extrapolated to the current rates of chronic stress and burnout documented in veterinary medicine. At worst, individual burnout precipitates turnover and loss of valued professionals, associated direct organizational recruitment and replacement costs, and spread of these effects through teams. The organizational cost of replacing a physician is estimated to reach 2–3 times the physician's annual salary (154). In the US, the impact of burnout in veterinary practice has been estimated to reach $2 billion in lost revenues each year, with a median cost of turnover of $104,000 for each veterinarian, and $59,000 per veterinary technician (85, 123).

Four types of reactions have been described in clinicians dissatisfied with their working environment: (1) “exit” (or intent to leave), (2) “voice” (the expression of job dissatisfaction by “speaking out” or taking some action such as unionization), (3) “loyalty” (taking an active role in changing the organizational causes of job dissatisfaction) and (4) “neglect” (a more passive approach that attempts to avoid the causes of job dissatisfaction) (117). Ultimately, clinicians experiencing unaddressed job dissatisfaction, burnout, anxiety, depression and/or PTSD generally either leave the clinical setting (through career changes within the institution), depart the organization for clinical positions elsewhere, reduce their clinical work hours, change specialty, or retire early, or leave the profession (117). Similar to the reductions in career choice satisfaction expressed by veterinarians (109), regretting one's career choice is three times more common in burnt out physicians than in their unaffected counterparts (OR = 3.49, 95% CI = 2.43–5.00) (120). While both personal and organizational factors play a role in decisions to leave a specific employer or the profession, management of burnout is an important aspect of veterinarian wellbeing and retention (155, 156). Burnout is one of the main factors influencing physicians' decision to leave their current practice (157, 158). On an individual basis, anxiety related to work-pressure and severe signs of burnout are predictive of turnover intention; the more severely affected, the more likely a professional is to quit (159). Physician burnout and satisfaction scores correlate with actual reductions in work hours over the 24 months subsequent to measurement (160). After controlling for age, sex, site, and specialty, each 1-point increase in the 7-point emotional exhaustion scale (OR = 1.43, 95% CI = 1.23–1.67, *p* < 0.001) and each 1-point decrease in the 5-point satisfaction score (OR = 1.34, 95% CI = 1.03–1.74, *p* = 0.03) were associated with a reduction in professional effort (160).

Extrapolating these data to veterinarians should raise concerns about the adequacy of the future workforce, considering the current care shortage in the veterinary profession (161). A veterinary health report by the Mars Corporation in 2021 predicted that the U.S. will need 41,000 more veterinarians by the year 2030, representing an anticipated shortage of nearly 15,000 veterinarians (including both general practitioners and specialists) and resulting in a lack of access to care for an estimated >75 million pets (162). Work hours reduction can be an effective strategy to reduce burnout for individuals but has important implications for the entire workforce. The burnout observed in US physicians between 2011 and 2014 was estimated to deplete the workforce by ~1% through shifts from full-time to part-time employment or departure from clinical medicine, an effect roughly equivalent to eliminating the graduating classes of 7 US medical schools (154). Extrapolating this effect to veterinary medicine does not offer much optimism about the potentially impending wider profession-level impacts of burnout, currently estimated to affect ~50% of veterinarians at varying levels of severity (1, 84). This concern is amplified in the context of the expected ongoing growth in the demand for veterinary care (97, 162–165).

## 5. Organizational prevention or reversal of burnout

**Figure 2:**
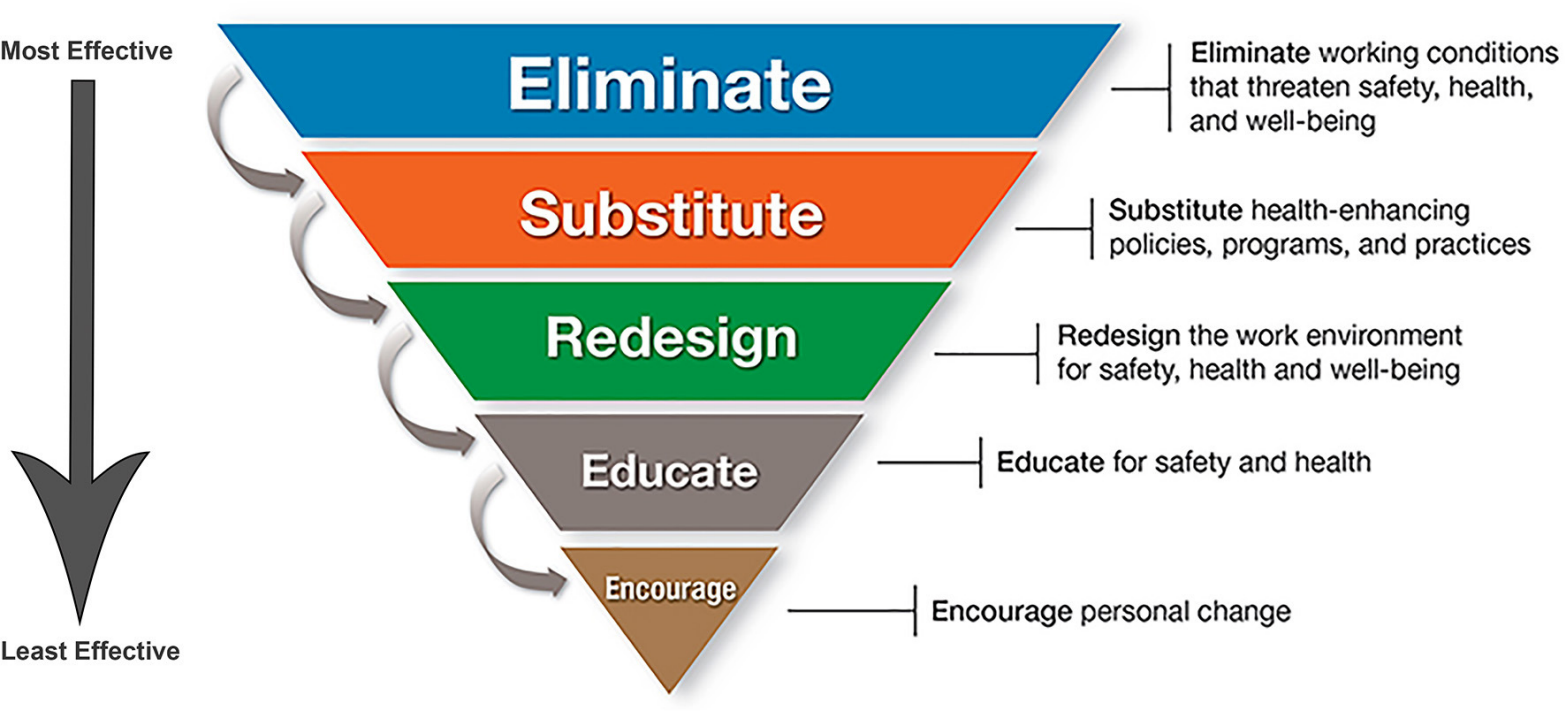
Hierarchy of Controls Applied to NIOSH Total Worker Health (170). An applied model of the traditional hierarchy of controls used in occupational safety and health that emphasizes organizational-level interventions to protect workers' safety, health, and wellbeing. Workplace programs using a total worker health approach should emphasize elimination (the top of the hierarchy) or control of workplace safety and health hazards as the primary goal. Hazards that cannot be eliminated must be managed through engineering, administrative, or, as the very last resort, individual-level interventions. Addressing environmental determinants of health rather than focusing on individual-level ones (the bottom of the hierarchy) is a crucial concept. This figure is minimally adapted from materials developed by the CDC. The unaltered material and additional resources are available on the CDC website (NIOSH TWH website) for no charge. Use of this material does not imply endorsement of this manuscript by the CDC or United States government.

Working in a veterinary practice organization with a healthy work culture is predictive of high wellbeing, low burnout, and an absence of serious psychological distress (109). It is easier and less costly in all ways for organizations to prevent burnout rather than to address it once established. There is an expanding literature on suggested improvements to the healthcare workplace targeted to improve wellbeing and reduce burnout, and a variety of opportunities for future discussion and suggested actions within healthcare education and practice have been identified (117, 166–169). The Hierarchy of Controls Applied to NIOSH Total Worker Health^®^ from the U.S. Center for Disease Control provides a conceptual model for prioritizing efforts to advance safety, health, and wellbeing in occupational settings (170). This hierarchy emphasizes organizational-level interventions to promote worker health and safety, and Figure 2 graphically depicts these steps from most effective to least effective (170).

Workplace stress and burnout should be anticipated as occupational hazards in healthcare and approached with a perspective of risk reduction. In order for risk reduction interventions to be effective, an intervention must be well-targeted to the underlying cause of the identified risk (171). As a risk, workplace stress is represented by both unavoidable occupational suffering that is inherent to the professional role, and avoidable occupational suffering that may be prevented or corrected at the organizational level (1). But even some types of stress that are fundamentally unavoidable may yet be better managed by improvement of workplace systems (172). For example, client complaints will never be completely eliminated as a practice stressor, and an individual veterinarian experiencing excessive stress from negative client interactions might consider whether methods of cultivating resilience (173, 174) might improve the associated stress. But just as importantly, an affected practice would benefit from evaluating its systems to identify whether there are logistical improvements (e.g., scheduling, wait times, and access to information) that can reduce client frustration and negative interactions with staff. Ongoing efforts by professional organizations improve public awareness regarding the many occupational stressors that veterinarians face may also help to improve client interactions. Even for unavoidable stressors, organizational attention and a combined, multi-pronged, well-targeted approach is likely to provide the best results.

Fundamentally, the primary manner a healthcare practice of any size supports its employees is by giving individuals firstly the ability to do their jobs with workflow and systems that foster teamwork, efficiency, and quality of care, and secondly by allowing them to return safely home with sufficient time and emotional energy to rest and engage in their personal lives with family, friends, and community (166). The specifics whereby this is accomplished will be different from organization to organization, and even from individual to individual. In the 2021 Merck Animal Health Veterinarian Wellbeing Study, four factors emerged that defined a healthy work culture. These included a strong sense of belonging to a team, a high degree of trust in the organization, candid and open communication among team members, and sufficient time allotted to provide high-quality patient care (109). Cynicism results when organizations lack effective communication strategies, and employees under these circumstances disengage from the work environment because they do not feel heard or considered (175).

### 5.1. Organizational support of individual solutions

Physical health is associated with psychological resilience. Given that veterinarians reportedly experience occupational illness or injury at a rate nearly three times that of physicians, policies that promote individual physical health, enable exercise, and improve nutrition may represent areas that could benefit from greater institutional support (4). Individual approaches in combating physician burnout include stress management and resilience strategies, counseling, coaching on self-care techniques such as diet and exercise, support for more autonomy at work, coaching on time management skills, and providing protected time away from work for family, friends and activities that bring joy and meaning to one's life (65). Targeted wellness programs developed for practicing clinicians that include training in cognitive coping skills and stress education, can enhance resilience in participants to a certain extent (176, 177). However, application of individual-targeted strategies to those in the midst of existing burnout is challenged by the affected individual's ability to focus on the recommended approaches in the face of other commitments and time constraints, and an individual's motivation or capability to follow through may be low as a result of existing stress and other responsibilities (39, 65). Additionally, the irony of many of these initiatives is that while they are organizationally coordinated, the onus of change is placed on the individual, with the implication that ongoing stressful occupational conditions themselves do not need to be addressed (178). The American Medical Association has established a blueprint that offers some ideas for mitigating healthcare burnout (179). Organizational support of personal resilience training is one of those steps, but it is the last in the list at step number 9, with steps 1–8 representing primary organizational action. While online wellbeing tools and resources (180–182) have been made available to veterinarians, only 12% of veterinarians have accessed such resources (183). Broad workplace wellness program participation is often limited across professions in part because of rigid work schedules (184). In order for these types of programs to have impact, employees must have sufficient time away from both home and work responsibilities to participate. Organizations can support clinicians in wellbeing efforts by providing protected time to participate in self-care activities, such as yoga, mindfulness training, and exercise without negative consequences to their career or advancement opportunities (65). While veterinary-specific data on the impacts of these types of interventions are limited, much like what is reported for physicians, academic veterinarians in a recent survey reported difficulty in attending institutionally offered wellness programming due to conflicts with their clinical duties or other work responsibilities (135). Attention to occupational spaces (185), including sufficient spaces for breaks and/or quiet retreat, organization that facilitates efficiency, productivity and collaboration, appropriate soundproofing, attention to workplace ergonomics for people of all sizes, access to natural areas and daylight, attention to workplace safety and security, on-site child care and/or personal pet spaces, and access to healthy food are other ways that an organization may also support individual stress management and wellbeing solutions.

On an individual level, veterinarians can help themselves and each other by becoming aware of the early symptoms of burnout, working to foster changes in professional culture, supporting improved boundaries between work and home life, and helping each other to find needed resources and assistance, whether that be by organizational change, mental health care, physical health care, a greater sense of community, or a combination of the above. Individual physicians are notably poor at recognizing when they are practicing unhealthy habits, and especially poor at addressing their own health problems once they are recognized (186). In addition to their direct responsibilities to their patients, veterinarians have a professional duty of care to guard against burnout in themselves and in their colleagues. In order to make progress on solving the issues of burnout, work stress, and mental and physical health in veterinary medicine, early recognition of the signs of professionals in distress is crucial. As symptoms are appreciated, recognizing when colleagues are approaching the point of cognitive overload, individual veterinarians can provide assistance and act as support.

### 5.2. Organizational solutions

Although each individual is ultimately responsible for their own wellbeing and individuals are encouraged to actively practice self-care, organizational awareness, attention to, and recognition of burnout and its underlying causes, plus commitment to address them are imperative to create significant change in profession-wide burnout statistics. Ongoing research to better evaluate interventions in the context of veterinary-specific situations and to determine optimal application timing may help to provide the greatest impact. On the whole, while interventions directed toward the individual and organization-level strategies can reduce burnout, the impact of organizational interventions is greater and potentially longer-lasting (3, 187). A resilient practice is one with systems and cultures that support its workers, and that can manage disruption and maintain normal function during both stable and crisis periods (166, 188). A veterinary practice seeking to reduce burnout and improve wellbeing should concentrate on fixing the workplace and creating a resilient organization, rather than fixing the employee (166, 189).

While evidence-based, veterinary profession-specific data is currently limited, and much work remains to be done defining what works best in different situations and veterinary practice environments. However, information is growing and there is also an expanding adjacent but relevant literature in human healthcare focused on organizational solutions to occupational stress. Useful texts targeted toward these issues represent an excellent starting point in applying these concepts to veterinary medicine (190–193). Occupational stressors in veterinary medicine have been described (1), and targeted areas for organizational improvement in the veterinary workplace are many and varied (Table 1). Strategic application of interventions will necessarily vary from individual workplace to workplace. In a recent survey of academic veterinarians, respondents desired more recognition and acknowledgment of employee workloads by leadership, improved collaboration between leadership and employees, more support and advocacy for employee wellbeing, more support for employees' personal, occupational, and financial needs, reduced clinic and teaching workloads, reduced administrative workload, offering more mental health days, and facilitation of direct pathways for employees to voice personal needs with leadership followed by genuine recognition and action (135). Factors identified by veterinarians in emergency practice as important in fostering a work environment conducive to long-term employment included leadership recognition of the value an individual brings to the practice, reasonable personal time off, receiving assistance when needed and being able to provide help when it is requested, and prioritization of a positive environment (108). Single-veterinarian practices have the benefit of being nimbler in their ability to implement changes that address burnout compared to larger organizations. Conversely, they may also have different subsets of stressors that require outside-of-the-box thinking to manage issues relevant to their individual practice situations, such as organized regional multi-practice affiliations to manage after-hours cases in a sustainable manner, or development and utilization of regional or on-line peer support structures. Success in making meaningful improvements will require time, resources, creativity, and patience, proportionate to the complexity of the organization (166).

**Table 1:** Targeted areas for organizational improvement of veterinary professional wellbeing (65, 97, 166–169, 190–192, 194–197).

**Organizational targets to improve veterinarian wellbeing**	
**Confront existing organizational issues:**	• Add measures of employee wellbeing to routine institutional performance assessments
• Acknowledge and reduce complexity of workloads where possible	• Reassess whether incentivization strategies undermine wellness efforts
• Acknowledge and reduce excessive workload volume	• Reassess whether incentivization appropriately targets the desired work focus
• Reassess performance expectations and reconfigure those that are unreasonably high	• Reduce bureaucracy
• Reduce excessive workday length/overtime, ensure work compression does not occur	**Promote workforce health and wellness:**
• Improve clinician autonomy and control over work	• Discourage presenteeism
• Identify and address systems inefficiencies and redesign workflow as indicated	• Align schedules to appropriately manage workload and patient care when staff illness occurs
• Assess technician/clinician/patient ratios and improve staffing where needed	• Integrate wellness, resiliency, and self-care skills into veterinary curricula and workplace settings
• Offer work flexibility where possible	• Offer work-life integration support
• Reassess scheduling for existing on-call and after-hours work systems. Hire additional staff and implement alternate scheduling as needed.	• Where relevant, acknowledge and offer aid with impacts of educational debt, low salaries and/or personal financial issues
• Account for other non-clinical workplace demands/pressures/expectations and their associated complexity of mental effort and logistics	• Incorporate the demonstration of health and wellness of students, faculty, and practitioners into academic accreditation requirements
• Improve staff access to administrative support	• Align supportive wellness policies
• Identify and mitigate causes of staffing turnover	• Offer a variety of opportunities and availability of health and wellness resources
• Address toxic work environments including bullying/mobbing	• Reduce workplace-related challenges in accessing health/wellness resources (e.g., logistical conflicts)
• Address resource hoarding	• Encourage time within the workday for wellness
• Remove time barriers to professionalism and teamwork	• Provide quiet space for breaks
• Address persistent historical negative institutional norms	• Eradicate stigma of experiencing burnout
• Assess and reconsider management styles and techniques; improve deficiencies in compassionate leadership, uphold organizational fairness and transparency	**Support students, trainees, and professionals in the practice setting:**
• Reduce technological burden where possible	• Acknowledgment of problems in the workplace environment by leadership
• Provide adequate equipment technological support	• Improve alignment of health professional skills and interests with work effort
• Address burdens of email and messaging systems	• Provide support during contentious client interactions
• Minimize complexity and improve efficiency and usability of medical record systems	• Organizational emphasis of the importance of health and wellbeing in both students and practicing health professionals
• Include a broad representation of clinicians and specialties in policy discussions	• Provide accessible education regarding individual wellness techniques
• Address salary inequities if present	• Take actions to reduce stigma associated help-seeking
• Consider perspectives of underrepresented professional groups	• Train and create positive role models
• Address conscious and unconscious biases in individuals and workplace culture	• Provide appropriate and adequate preparation and support for trainee mentors

### 5.3. Intervention areas

#### 5.3.1. Workload and job demands

Some of the most consistently mentioned concerns of veterinarians and other healthcare professionals in association with burnout, job satisfaction, and intent to leave are workload and time pressure (108, 117, 132, 135, 198–201). Recommendations for mitigating physician burnout include reducing workload and providing a suitable working pace to reduce cognitive burden (202, 203). Workload may be cognitive or physical in nature, and it is increasingly recognized that the complex nature of modern medical workloads may be just as important as actual work hours. A dose-response relationship between cognitive load (represented by task load) and burnout has been reported (204). Caseload expectations should be set based on reasonable human cognitive load limits and what is required to provide good patient care. Manageable case numbers will be higher in less complex caseloads and lower in more complex caseloads. If case complexity represented by task load per case has risen, the time required to provide good patient care has also risen per individual, and what historically used to be a reasonable inpatient census per clinician may no longer be viable. Outpatient scheduling with timing that appropriately reflects case complexity and considers the overall task load of the case type seen should also be considered. Medical workloads in general need to be re-evaluated and redesigned with realistic expectations in line with human cognitive, emotional, and physical limitations, with accompanying organization-wide training and management support (205, 206). Altered practice caseloads will have impacts on staffing, budgets, and training, and solutions are neither simple nor universal and must be evaluated in the context of each individual practice environment. However, while arguments for excessive workloads and job demands as solutions for practice challenges may be strong from a practice economic and logistics standpoint, the preponderance of scientific evidence simply does not support such allowances. The evidence that time pressure and heavier workloads on clinical personnel result in a variety of poor outcomes critical to the cost and quality of care should incentivize practice leadership to act proactively to minimize clinician burnout (117). Practices and organizations should track dissatisfaction and early signs of burnout as indicators of practice/health system dysfunction that require changes in the immediate to near future (207). Research on methods of improving practice efficiency most relevant to veterinary-specific environments would be useful to inform the specifics of future recommendations (e.g., use of scribes or scribing software, simplified medical records systems, automating or delegating systems of medication refills, use of veterinary technicians with advanced training or development of novel veterinary paraprofessionals similar to physician's assistants for certain caseloads, evaluation of case flow and different appointment or procedural scheduling mechanisms especially in larger/complex hospital environments, among others).

#### 5.3.2. Work complexity and operational efficiency

Across professions, burnout is linked with high work complexity as well as total workload, so it logically follows that managing burnout must include improving system complexity at all levels (e.g., for individuals, teams or subgroups, and the organization as a whole) (205). Low predictability within job duties requires clinicians to process large amounts of additional information quickly, and forces deviations from expected trajectories; the more chaotic the environment or workflow, the greater the impacts on performance and stress (134). Although impacts of burnout on individuals may lead to medical errors, not all errors are attributable to burnout, as a burnt-out workforce may spotlight a system already prone to dysfunction and resultant errors (118). The most vigilant and careful clinicians will be especially prone to burnout and withdrawal if frustrated by a dysfunctional system; these are likely to be the ones the organization should want to retain most. Clinician burnout may be the best early indicator of serious system dysfunction, before significant errors occur (118). This may be particularly important in larger institutions, impacting veterinary academia but also private practice, given the ongoing shift in the profession from smaller, individually owned practices, to larger, complex, and corporate practice.

At a certain point, being able to provide consistent clinical excellence in healthcare depends on operational efficiency. Between 2017 and 2021, at least 60% of companion animal practices exhibited severe inefficiency problems (113). When the gulf between system capacities and patient needs is too wide or occurs too often, the impossibility of serving both imperatives contributes to moral distress and burnout (166). Business concepts such as continuous quality improvement and workflow efficiency may help generate ideas for improvement where relevant, with the goals of improving patient flow, scheduling, efficiency, and staff satisfaction (117, 166, 208). However, too much standardization creates its own challenges and overall, most clinicians respond better to an approach of “empower and encourage,” rather than “command and control” (166). Combination approaches that reduce job demands, reduce complexity and unpredictability, improve job resources, improve clinical workflows, enable relevant work autonomy, and provide support in ways specific to each unique organization are likely to be most effective (166). Ensuring that all team members are recognized for their contributions enhances individual professional efficacy (135, 175). Workflow, mental load, and efficiency can be improved by seeking an optimized balance between individual customization (too much of which can be chaotic, time-consuming, unpredictable, and unreliable) and standardization (too much of which can be oppressive, disrespectful, and prevent adaptation) (166). This balance will be different for each unique practice environment. Finding a better balance between these approaches permits clinicians to spend their finite cognitive bandwidth and emotional energy on the situations that require their expertise, and this reduced mental load contributes greatly to professional satisfaction (166).

#### 5.3.3. Organizational management, culture, and climate

The practice or educational organization must also provide a culture and climate that reduces stigma and embraces and facilitates burnout recognition, prevention, and management. At a minimum, all organizations should seek to foster systems that minimize burnout risks. But a professional culture that regards chronic stress as a rite of passage promotes judgment and feelings of self-stigma when burnout does occur (when individuals believe that they should be able to cope with this supposedly unavoidable aspect of their chosen career) and leads to worsening stress and reduced help-seeking (209). “*Fundamentally, many leaders are mismanaging some of the most talented professionals in their health care delivery systems and organizations*” (210). When burnout occurs, management should support and encourage professionals (who may be averse to appearing imperfect or vulnerable) to discuss workplace stress concerns and experiences without fear of reprisal or stigmatization, and to foster mechanisms whereby help is provided (118). Suggested processes for such programs can be extrapolated from published concepts for helping physicians suffering from other forms of impairment (211). It is imperative to create an occupational environment where clinicians are comfortable speaking up as well as empowered to develop work-life balance and seek assistance when they need it. Wellness-centered leadership has been proposed to empower individual and team performance in human healthcare environments (210). Explicit training for practice managers, practice owners, and academic leadership on burnout and other occupational health issues is important and necessary.

Rather than wait and respond reactively to clinician burnout, human healthcare is increasingly recommending annual, proactive, institutional checkups on the wellbeing of the professional workforce whereby clinician wellbeing is measured and monitored using wellness metrics, observation of withdrawing behaviors or their adverse effects, or identification of the presence of stressors via surveys (75, 169, 193, 212, 213). However, in order for workplace efforts to reduce burnout to be effective, it is fundamentally necessary for veterinarians and trainees to have confidence not only in the given interventions, but also in those who are delivering them (194). More than 75% of the key reasons across professions that employees quit are heavily influenced by management techniques and choices (214). Managers seeking to reduce turnover must pay close attention to which influence tactics they use to lead their employees, promoting inspirational appeals and minimizing pressure tactics whenever possible (214). The impacts and importance of organizational leadership was highlighted in a recent veterinary study (97).

#### 5.3.4. Team environments and workplace bullying

Optimizing team environments often requires monitoring for the very wide range of situations that workplace bullying may manifest, from overt actions (e.g., threat to professional status, destabilization, isolation, targeted overwork, verbal taunts, or violence), to more covert actions (e.g., targeted incivilities, exclusion, gossip, verbal criticism, insults, mimicking, name-calling, intimidation, withholding information, unfair assignments, allocating undesirable tasks, or sabotage) (215, 216). From an organizational perspective, bullying reduces employee motivation, commitment, creativity, and productivity, and increases employee turnover (217). Injustice and unfairness are predictors of aggression in the workplace, resulting at times in those individuals who experience bullying to subsequently exhibit disruptive behavior themselves (1, 136, 218). Indeed, poor management has been cited as the greatest risk factor for the occurrence of workplace bullying (219). However, much like burnout, the majority of current strategies for addressing and preventing workplace bullying (such as anti-bullying policies, bullying awareness training, and incident reporting and complaint investigation) focus primarily on behaviors between individuals and commit the critical error of overlooking risk factors in workplace structures (219, 220). Maslach et al. recommend focusing interventions for bullying/incivility at the work unit level, rather than an individual level as the most effective strategy (195), although egregious individual issues will certainly need to be addressed directly. Encouraged qualities representing workplace civility include being attentive to colleagues, listening to their views and concerns, accommodating one another's preferences, and anticipating the impact of one's behavior on others (195). It is recommended that veterinary practices actively monitor the effectiveness with which their teams operate, carefully evaluate whether organizational assessment and reward structures reduce or exacerbate individualistic vs. team role conflicts, provide non-judgmental mediated assistance to work units that are struggling, and overall, appropriately incentivize highly functioning teams (221).

#### 5.3.5. Poorly designed incentivization strategies

Both absolute salary level (insufficient reward) and relative salary discrepancies (lack of fairness) can be sources of stress or professional dissatisfaction leading to burnout (116). For some clinicians, increased take home pay may help improve burnout through additional funds that help with home chores and by doing so, free up personal time. Indeed, lower burnout scores were identified for veterinarians in higher income categories, beginning at annual salaries of $150,000 (85): for many, this likely reflects alleviation of financial stress relative educational debt as having a higher salary improves the ability to meet monthly loan payments. Relative salary differentials may also be symptomatic of organizational inequities (222), which may be yet another contributor to higher rates of burnout in women than men, given the gender pay gap (89, 91). However, for individuals (e.g., house officers) who are at higher risk for burnout as a result of serious financial limitations relative to costs of living, increasing take-home pay is the most obvious, just, and urgent solution (40, 56, 223). Increased resident income is associated with increased career satisfaction (34, 183).

Productivity-based compensation as an incentivization structure has been associated with increased burnout risks (1, 224). Studies of behavioral economics have shown that monetary rewards can undermine motivation and worsen performance on cognitively complex and intrinsically rewarding work, suggesting that pay-for-performance in healthcare may actually backfire (225). Suggestions include consideration of alternative compensation models in which productivity incentives are targeted toward alternative rewards (increased time off, greater schedule flexibility), consideration of performance-independent salary models, incorporation of structures that reward quality-based measures, or incorporation of measures of clinician wellbeing into performance assessments (224).

#### 5.3.6. Job control, autonomy, meaning-in-work, and camaraderie

Some occupational stress theories place emphasis on increasing individual job control/autonomy as a mitigator of work stress leading to burnout; improving autonomy has been suggested to improve burnout in physicians (34, 226). In one study of 608 physicians, the single most powerful predictor of burnout was a low sense of control over the practice environment (*p* < 0.05) (227). It should be noted that job control will not ameliorate the independent effects of excessive work hours and demands, however when high work demands are already present, burnout worsens when clinicians have reduced control of their schedules (228). Flexibility in work assignment, matching work to the talents and interests of individuals, increasing opportunities for professional development, increasing organizational engagement, and promoting camaraderie may also help to prevent burnout (229). Seventy to 75% of veterinary student and practitioner respondents in a recent survey targeting the shortage of veterinarians in emergency practice indicated that a flexible work schedule would encourage them to enter or remain in the field of emergency medicine (230). Evidence in physicians suggests that spending a minimum of 20% of professional effort in the aspect of work that is most personally rewarding may be the necessary threshold to reduce burnout and maximize work satisfaction (144). However, many of the burnout models that emphasize autonomy also undervalue hospital culture, climate, and efficiency issues as variables and ignore that job control does not protect employees from generally poor working conditions (29). Increasing work control and time autonomy were not associated with burnout improvements in healthcare workers in other studies, and prioritizing interventions to mitigate and cope with constantly growing workloads, excessive hours and unsocial work schedules was recommended (231). Promoting perception of the meaning in work has been proposed to reduce burnout in human healthcare (232), and healthcare professions are commonly described as being “a calling.” The work-as-calling theory does associate the perception of a professional calling with positive work-related outcomes (e.g., increased job satisfaction and job performance) under some circumstances, but also may lead to negative outcomes (e.g., workaholism, burnout, and workplace exploitation) under others (233). Encouragement of physician solidarity reduces burnout, so organizational policies and infrastructure that encourage and support peer interaction and peer support may be helpful in mitigating veterinarian burnout (135, 224). Where possible, implementing means to increase clinicians' autonomy, job control, organizational engagement, camaraderie, and mindful approach to practice, as well as emphasizing recognition and acknowledgment of their efforts by leadership are positive steps in a multi-pronged approach that are encouraged where feasible and relevant, but are not a replacement for needed improvements in fundamental work overload and dysfunctional work systems where these types of problems exist (232, 234).

#### 5.3.7. Presenteeism

Between 54 and 99% of veterinarians have indicated that they would come to work despite the presence of symptoms consistent with infectious illness, consistent with reports of sickness presenteeism in human healthcare (235). Reduction of presenteeism behaviors and cultural shifts will require efforts from individuals as well as healthcare organizations. However, this cannot be accomplished if organizational policies regarding disease prevention behaviors are equivocal, if systems incentivize staff to work when ill, or if practices do not staff sufficiently so that clinician illness can be accommodated, and workload redirected in a functional manner when clinician illness inevitably does occur (235, 236).

#### 5.3.8. Occupational sleep restriction and insufficient rest and recovery

Prolonged work hours due to deliberate scheduling of extended shifts or overrunning of standard workdays due to practice inefficiencies, high workload, and extended workweeks, as well as occupational sleep restriction resulting from interrupted sleep while on call, insufficient recovery sleep afterwards or circadian disruption from poor scheduling, all lead to insufficient rest and lack of physiologic recovery, a fundamental contributor to burnout (237–239). Improving sleep can lessen the negative association between stress and clinician functioning (55). On-call duties (especially involving shifts of ≥24 h) are consistently associated with high levels of clinician stress and greater burnout risks; unsurprisingly progressively fewer clinicians in both human and veterinary medicine are willing to take on-call duty as part of employment (66, 97, 124, 131, 240, 241). Given what is known about the physiologic impacts of sleep deficit on human functioning and the importance of sleep to burnout recovery, reduction of occupational impacts on clinician sleep is absolutely fundamental to reduce the occurrence of and to mitigate existing burnout (52, 54, 202, 203, 238, 242). This includes attention to daily and weekly duty hours to permit sufficient time for rest and recovery, as well as urgent attention to working arrangements and scheduling to improve circadian alignment in after-hours work shifts (202, 203, 238, 242, 243). However, in order to see positive effect when addressing these issues in duty hour schedules, organizations must take care that changes do not result in worsening work compression and increased cognitive burden (44, 206).

### 5.4. Veterinary education

The doubling time for knowledge in 1950 was an estimated 50 years, but it accelerated to 7 years by 1980, to 3.5 years by 2010, and was projected to be 73 days by 2020 in a 2011 study (244). Veterinary education increasingly emphasizes self-directed learning skills (245) and to manage this information growth, the technology needs of veterinary education and practice may ultimately increase in the form of computerized clinical decision support software [such as Elsevier's ClinicalKey^®^ for physicians (246)] and artificial intelligence systems (247–249). Given the exponential expansion of scientific knowledge in modern medical practice, at some point, the profession may need to re-evaluate the fundamental structures and expectations of veterinary education and/or potentially consider future limitations or specifications on different types of veterinary licensure. While controversial, accelerated 3-year tracks are offered in some U.S. and Canadian medical schools with the goals of reducing student debt and more quickly addressing physician shortages (250).

The training of future veterinarians, scientists, and specialists takes place predominantly in academic institutions. Strategies to reduce trainee burnout should focus on overall workload, as well as curricular structure and delivery (251). Addressing veterinary burnout should begin preventatively in the first semester of veterinary school. On an individual level, improved sleep, better nutrition, more exercise, and more frequent contact with one's support system predicts improved psychological wellbeing in veterinary students (252). Veterinary school is also the optimal time for providing resilience training and skills, and real education about the concepts and specific techniques of wellbeing and sleep adequacy as aspects of professionalism, the concepts of presenteeism and burnout, and how these types of choices affect both one's person and one's professional efficacy and longevity (253). Professional students who are taught mindfulness describe decreased perceptions of stress, anxiety, and depression, and exhibit increased mindfulness, empathy, and self-compassion (254, 255). Behavioral change plans have been reported to be valuable and effective exercises in a cohort of 2nd year medical students, enabling them to practice the strategies and experience the obstacles of changing health behavior, and after completing the assignment, 80% of participating students considered themselves to be healthier (256). However, internalization of these concepts and behaviors by students does require that academic mentors actively and legitimately model and support self-care and professional balance.

Reframing veterinary culture and what represents the norm should occur early. Retraining veterinarians in how they conceptualize professionalism and professional wellness after they have completed training is more challenging, as maladaptive behaviors have already become deeply embedded. The recent 2017 revision of the Declaration of Geneva (the contemporary successor to the physicians' Hippocratic Oath) contains the words: “*I will attend my own health, wellbeing, and abilities in order to provide care of the highest standard*” (257); veterinary medicine would do well to follow this lead. Similar to the issue of veterinary debt, addressing the issues of professional stress and stress management even earlier than veterinary school itself via pre-veterinary college curricula might help prospective veterinary school applicants to: (1) better plan and manage their educational trajectories and build resilient habits earlier in their careers, and (2) improve financial literacy before veterinary school [a source of a great deal of chronic stress in students, house officers, and early career veterinarians and physicians (40, 59, 60)], and (3) allow them to make more informed decisions about their intended career paths before they commit themselves to a trajectory that may not meet their initial perceptions or expectations, rather than expect them to opt-out once emotionally, logistically, and financially committed. Opting out at that stage is highly disincentivized by perceptions of failure and stigma, as well as the magnitude of unrecouped costs of veterinary school from a truncated career.

Poor mental health or self-care among residents and their faculty mentors undermines the student experience, emphasizing the importance of also improving the mental health of the residents and faculty with whom students work (258). A top-down approach to burnout is important because it sets minimum standards early in training (259). Veterinary house officers clearly identify their experiences with burnout as direct effects of their working conditions and program characteristics rather than because of personal failure to manage stress or make balanced lifestyle choices (50, 260). Teaching resiliency and self-care and promoting clinician social support alone will not suffice; more fundamental reform is required. The American Council of Graduate Medical Education, which is responsible for physician residency accreditation, has acknowledged the growing problem of physician burnout by adding a “wellbeing” requirement for all residencies (261). In veterinary medicine, the most urgent house officer issues to target are overall workload, work hours expectations, and scheduling that leads to sleep insufficiency, with additional immediate attention to house officer salary inequities (40, 50, 56, 260).

Reduced patient load is associated with increased physician resident satisfaction and time spent on educational activities, but also does result in cost increases of hiring additional staff (262–264).While acknowledging the economic impacts and management hurdles, it has been suggested that to accomplish goals of reducing physician resident workload, resident position numbers should be increased to reduce work intensity, and that in settings of high work intensity, clinical service burden should be shifted from residents to non-resident providers (265). An experimental restructuring of an inpatient internal medicine service documented improved resident physician and medical student experiences and a favorable impact on patient outcomes after acting to reduce the per physician patient census (266). Another service model with a unit-based admissions process in which patients and care teams were consolidated within hospital units and inpatient census caps were instituted on resident-based services reported improved mean resident ratings of workload appropriateness [3.10 (SD = 0.08) vs. 3.87 (SD = 0.08) on a 5-point scale; *p* < 0.001], improved rounds attendance [1,523 (57%) vs. 1,700 (64%), *p* < 0.001], and reduced duty hours violations [from 70 (SD = 0.74%) to 14 (SD = 0.15%; *p* < 0.001)] (263). Increased resident numbers were associated with improved resident physician satisfaction of overall program quality, improved rounds quality, and improved discharge summary quality (266, 267). Reducing resident workload by addition of non-resident staff was associated with improved perceptions of the educational value of the program, levels of responsibility and supervision, and a “just right” level of patient volume while maintaining an appropriate caseload variety (44).

While urgent and necessary, trainee work reform has also been shown to have serious impacts on the professional lives of faculty mentors (268). As a result of resident work hour limitations, physicians commonly report less time for research, teaching, and other academic pursuits (268). Increased patient:clinician ratios are associated with a greater perception that time for patient care is insufficient, increased faculty occupational stress, more faculty burnout, more negative attitudes among faculty regarding time for teaching, and reduced trainee satisfaction (133). These reported changes were associated with the self-reported probability of faculty physicians leaving academic medicine in the subsequent 3 years (268). Veterinary student and house officer work reform is morally and temporally urgent but must be accomplished without promoting work compression or further worsening faculty stress and risks of burnout in order to avoid exacerbating the baseline challenges in recruitment and retention of clinical specialists that often exist within veterinary academic medicine.

## 6. Future work on veterinary burnout

As further research is performed on impacts and solutions for veterinary professional burnout, as in the human healthcare literature, careful attention to study design is recommended, with appropriate match of assessment instruments to hypotheses, and complete stratification of demographic data. Given the identified overlap in depression and burnout identified in health care providers, it has been recommended that valid depression scales also be employed when assessing for burnout (269). If the profession is truly trying to address different mental health concerns in veterinarians, it is critical to differentiate between them while recognizing the significant overlap among them. For example, burnout, moral injury, secondary traumatic stress, and mental health conditions such as depression, anxiety, and PTSD all exhibit overlapping manifestations, and may even coexist among some individuals, but they are separate and distinct conditions and require different interventions. Mitigation of burnout requires culture and system changes, moral injury and secondary traumatic stress benefit from efforts to improve resilience and stress coping mechanisms, whereas mental illness requires medical treatment. Additionally, within the existing literature there is heterogeneity in how burnout and quality of life are defined, as well as an overall lack of accounting for longitudinal variation of severity in burnout and quality of life fluctuations over time in individual subjects. There are a variety of different assessment tools for burnout and wellbeing, however the assessment tools are discordant and lack a central characterization for burnout, and a single tool may not cover the full spectrum of burnout indicators; this also makes comparison between different methodologies difficult (65, 270, 271). Also, questionnaires alone may not differentiate well between true burnout and short-term stress; individuals with short-term stress do also show elevated levels on burnout measures (153). It has been recommended that burnout data be analyzed via latent profiles to provide a more nuanced view of burnout, to allow for earlier recognition of workplace issues, and to facilitate more meaningful interventions and comparisons across populations (272). Many studies of burnout are designed to be measured at an individual level, despite the fact that most causes of burnout are informed by the organizational environment and are outside the individual's control. Much of the generated data on individuals does not provide actionable recommendations, which is ironic and problematic for what is a workplace problem that much of the time fundamentally derives from a lack of self-efficacy.

## 7. Discussion

Workplace stress in veterinary medicine has a serious and direct toll on productivity, efficiency, quality of care, and human capital. Veterinarians are commonly hard-working, passionate, altruistic, and patient-driven professionals, but existing paradigms are unsustainable for many. Excessive workplace stress and unsustainable working conditions have been such a strong component of veterinary practice for so long that culturally the profession tends to view it as normal, which contributes to the perpetuation of the problem. A host of rationalizations (economic, educational, etc.) are frequently offered by both practice management and clinicians themselves to support the necessity of systems as currently configured. However, these conditions can only be seen as acceptable and necessary if the health and wellbeing of clinicians are not a priority. Ultimately the health effects of workplace stress and burnout are approximately as great as the health impacts of secondhand tobacco smoke, a health risk deemed sufficient to merit widespread intervention (184). The cultural expectation or even veneration of long work hours and sleep deprivation in human and veterinary medicine would be considered unprofessional behavior (and even illegal) in other types of workplaces (273). The work-life balance of veterinary trainees is too commonly incompatible with satisfactory maintenance of personal wellbeing, yet the culture and habits internalized during school and postgraduate training set patterns of work behavior that persist long after. Burnout appears to be an important mediator in understanding suicidal rates and tendencies (274). Organizational interventions and coping resources effective in reducing burnout may also reduce the risk of suicide (274). A fundamental limitation of this review is the current relative dearth of well-stratified, comprehensive, veterinary-specific data in this area, and the direct extrapolation of the fine specifics of many recommendations from human to veterinary medicine is complicated by differences in workforce shortages and economic landscapes. However, on the basis of the existing information and contexts discussed in these reviews, despite the lack of profession-specific data in many areas, veterinary management inattention to the workplace-related root causes of burnout is unjustifiable.

If initiatives to reduce burnout and improve health within the veterinary profession are to be effective, they cannot simply advocate for symptom reduction and durable change is imperative. Improving veterinarian wellbeing will be most successful not by admonishing individual veterinarians to be more resilient, but by creating more resilient veterinary schools and practice environments that prioritize workforce wellbeing, regularly assess organizational burnout and share accountability for these outcomes across leadership roles, intentionally measure and improve the efficiency of the work environment and create a culture of support for clinicians (166). While prioritization of personal wellbeing is ultimately incumbent upon each individual veterinarian, it has been clearly demonstrated that a preponderance of the drivers of burnout are characteristics of the work environment, and that serious efforts to redesign, reduce or eliminate negative workplace and educational practices will be required for fundamental impact. Clinician, trainee, and staff wellbeing should be benchmarked and actively monitored and must be recognized as a missing but imperative quality indicator for all veterinary practice systems. Logically, application of the discussed principles will vary according to practice size, type, and setting, and not all concepts or ideas will apply equally to all organizations, but it is incumbent on practice and educational structures to ensure that employees and trainees are not required to function in environments that promote burnout and other adverse sequelae such as career exit, mental illness, and suicidal ideation in such high rates in veterinary medicine.

## Author contributions

MS conceived and designed the review and wrote the first draft of the manuscript. All authors contributed to analysis and interpretation, critical revisions for intellectual content, and read and approved the submitted version.
